# Long-Acting Injectable Prep for People Who Inject Drugs at a Syringe Services Program: A Qualitative Acceptability and Feasibility Study

**DOI:** 10.1007/s10461-024-04598-3

**Published:** 2025-01-09

**Authors:** Edward Suarez, Hansel E. Tookes, Marissa Coppola, Marina Plesons, David Serota, Sara M. St. George, Tyler S. Bartholomew

**Affiliations:** 1 Department of Psychiatry and Behavioral Sciences, University of Miami Miller School of Medicine, Miami, FL USA; 2 Department of Medicine, University of Miami Miller School of Medicine, Miami, FL USA; 3 Department of Public Health Sciences, University of Miami Miller School of Medicine, Miami, FL USA

**Keywords:** People who Inject Drugs, Syringe Services Programs, HIV, PrEP

## Abstract

Although people who inject drugs (PWID) are at high risk of acquiring HIV, knowledge and uptake of pre-exposure prophylaxis (PrEP) for HIV prevention among this population remain low due to numerous psychosocial and structural barriers. Multiple implementation strategies have been proposed to address this gap, notably providing long-acting injectable (LAI) formulations of PrEP and offering PrEP at syringe services programs (SSPs). This qualitative study explores the acceptability and feasibility of offering LAI-PrEP for PWID at risk for HIV at Florida’s first legal SSP. In-depth semi-structured interviews were conducted with PWID (*n* = 25) and healthcare providers (*n* = 5), and transcripts were analyzed using iterative thematic analysis. The provision of LAI-PrEP at the SSP was overwhelmingly acceptable to both PWID and providers, and specific advantages and disadvantages of LAI-PrEP compared to oral PrEP among this population were elucidated. Likewise, PWID and providers identified facilitators and barriers to offering LAI-PrEP at the SSP and proposed recommendations for implementation. Overall, this study adds to the growing evidence that provision of LAI-PrEP at SSPs is acceptable and feasible and holds promise in expanding access to and uptake of HIV prevention services among PWID.

## Introduction

Injection drug use has led to multiple recent outbreaks of HIV in the United States (U.S.), creating a challenge for achieving the *Ending the HIV Epidemic* (EHE) initiative’s goal of a 90% reduction in incident HIV infections by 2030 [1, 2]. Less than half of the people who inject drugs (PWID) living with HIV in the U.S. are virally suppressed due to a myriad of structural and social challenges impacting linkage to care, retention in care, and access to antiretroviral therapies (ART) [3, 4]. PWID experience greater challenges in remaining engaged in HIV care compared to other populations, with Hispanic and Black individuals being particularly affected [5]. The barriers to HIV care that PWID experience, including syndemic substance use disorders, mental health disorders, stigma, financial and housing insecurity, and lack of transportation, contribute to adverse health outcomes [6, 7].

Although pre-exposure prophylaxis (PrEP) is a CDC-recommended intervention and has been available since 2012, there continues to be a general lack of awareness among PWID of PrEP as an option to prevent HIV transmission [6, 8–10]. New implementation strategies are required to reach this population [11], and one particularly promising strategy is to offer PrEP at SSPs. There is a growing body of science suggesting SSPs are a promising location to implement health services where PWID can address syndemic health problems [8, 12–19].

The latest advancement in HIV prevention available to the public is long-acting injectable cabotegravir (LAI-PrEP), which is a formulation injected by a medical professional once a month for two months, then every other month thereafter (Bazzi et al., 2022). Results from HIV Prevention Trials Network (HPTN) 083, which compared LAI-PrEP to daily oral tenofovir/emtricitabine (TDF/FTC) among 4,570 cisgender men and transgender women who have sex with men in seven countries across the world, showed that LAI-PrEP was non-inferior to daily oral PrEP [20]. As part of comprehensive HIV prevention strategies, all forms of PrEP play a crucial role in efforts to end the HIV epidemic [21]. Although, LAI-PrEP is seen as a vital tool which may improve equitable access to PrEP and reduce HIV incidence, this population continues to be underrepresented in biomedical HIV prevention research [8, 13, 22, 23].

Qualitative studies suggest PWID who are more socially and structurally vulnerable to HIV prefer LAI-PrEP over oral-PrEP, and reducing barriers to PrEP uptake is vital (Eger et al., 2023; Bazzi et al. 2023; Biello et al., 2019). Implementation science research examining the acceptability and feasibility of LAI-PrEP among PWID has been conducted in a small number of studies in select locations [7, 13, 22, 24]. However, studies on LAI-PrEP and PWID are missing a perspective: voices from the South. SSPs capable of providing low barrier access to medical care could be well positioned to provide meaningful HIV prevention interventions, including LAI-PrEP services. Furthermore, LAI-PrEP embedded within an SSP equipped with staff who can provide resources to alleviate psychosocial and structural burdens could have the ability to decrease HIV incidence among this population. The purpose of this qualitative study was to build on this emerging body of evidence by exploring the acceptability and feasibility of offering LAI-PrEP for PWID at risk for HIV at Florida’s first legal SSP.

## Methods

### Human Subjects

The study was approved by the University of Miami Institutional Review Board in March of 2022 (IRB# 20220127).

### Study Design & Procedures

This study consisted of in-depth semi-structured interviews with PWID at risk for HIV who utilize the SSP’s services (hereafter referred to as “clients”) and clinicians who provide healthcare services at the SSP (hereafter referred to as “providers”) to understand their perspectives regarding the feasibility and acceptability of offering LAI-PrEP at an SSP for PWID. For the purpose of this study, acceptability refers to the extent to which a new intervention is perceived as appropriate, agreeable, or adequate to the people and communities it aims to serve. Feasibility refers to **e**xtent to which a new intervention is perceived as appropriate, agreeable, or adequate to the people and communities it aims to serve.

### Study Setting & Participants

All participants (clients and providers) were recruited using a convenience sampling method at the SSP between June 2022 and March 2023; clients were approached in-person with information about the study and providers were approached via phone or email. Clients were eligible for inclusion if they: (1) were 18 years of age or older, (2) were enrolled in the SSP, (3) had a non-reactive point-of-care HIV rapid test, (4) were currently taking oral PrEP, and (5) were able to speak and understand English. Providers were eligible for inclusion if they were involved in the provision of healthcare services at the SSP. All participants provided informed consent and received compensation of $50 USD for completing the 30–60 min interview.

### Data Collection

The interview guide for clients addressed the following topics: (1) experience taking oral PrEP, including any personal and/or structural barriers, (2) knowledge of and attitudes towards LAI-PrEP, and (3) recommendations for designing an implementation strategy to maximize client engagement (e.g., physical location, hours of operation, duration, frequency, provider characteristics) (Table 1). The interview guide for providers addressed the following topics: (1) barriers faced by PWID in accessing and adhering to oral PrEP, (2) attitudes towards LAI-PrEP for PWID, and (3) recommendations for designing an implementation strategy to maximize client engagement (Table 2). All interviews were conducted in English in a private location. The interviews were audio-recorded and transcribed verbatim by a third-party transcription service. Field notes were also taken by the interviewer. No repeat interviews were conducted. Demographic information is provided in Tables 3 and 4.

**Table 1:** Qualitative interview questions with clients

Topics Addressed	Sample Question
Advantages of LAI-PrEP	First, I would like to talk to you about what you might think would be some advantages to LAI-PrEP. • Have you ever taken oral-PrEP in the past and what was your experience? • Is this your first-time hearing about injectable PrEP? What have you heard? Probe: What interests you about it? • Do you believe it would ease the burden of remembering to take the medication and carrying it around or storing it? Probe: How would LAI-PrEP decrease the burden of daily medications management for you? Probe: How would LAI-PrEP decrease the burden of daily medications management for PWID? • What do you think would be the advantages to injectable PrEP in general?
Disadvantages of LAI-PrEP	Next, I would like to talk to you about what you might think would be some disadvantages to LAI-PrEP. • What concerns you about it? Probe: How consistent do you feel you would be if you had to get the shot every 2 months? • What might make you not want to take injectable PrEP (vs. oral PrEP pills)? Probe: relationship status, perceived risk, change in behaviors, fear of injection • After the medication has been injected into your body, it will stay in your system for at least two months. How do you feel about this? Any concerns? Probe: Have you had injectable medications of any kind delivered in the past? Probe: Do Side effects concern you or interactions with other substances concern you? • What do you think would be the disadvantages to injectable PrEP in general?
Facilitators to LAI-PrEP	What would be some of things that might encourage a PWID to get LAI-PrEP at an SSP? • Have you been offered oral PrEP by staff at [the SSP] in the past? How was that experience? Probe: did we find you on outreach?, or did you come to the main site?, how long did you stay on the medication? • How would you feel receiving this shot every two months at [the SSP]? Probe: What do you think is the best way to make sure the people who use [the SSP] get their shot every two months? • How do you think other PWID would feel receiving this shot every two months at [the SSP]? • How do you think the experiences gained injecting substances might influence how you think about getting shots of PrEP instead of pills?
Barriers to LAI-PrEP	What would be some of the things that might prevent PWID to get LAI-PrEP at an SSP? • What would be some of the things that would make delivery of LAI-PrEP difficult at [the SSP]? • What do you think other PWID would say if they knew you were receiving LAI-PrEP at [the SSP]? • Where else would you be willing to receive a shot of LAI-PrEP every two months if you did not want to do it at [the SSP]? • If the injectable is missed or delayed, you would be at risk of HIV infection, and if you got HIV during that time soon after stopping injection, there is a risk for the virus to become drug resistant. What are your thoughts on this? • If someone stops or delays the injections, they will have to use alternative HIV prevention methods (condoms, oral PrEP, abstinence). What are your thoughts on this?
Service Delivery Preferences	Let’s say that the SSP would begin offering LAI-PrEP as a part of its services. • What do you think would make PWID want to use this? • If given the choice, would you select taking one pill each day or a shot that is given once every two months? Probe: Why? Tell me more about why you made that choice. • Speaking for yourself, would you be able to come to [the SSP] for the shot at set times every two months (e.g. a scheduled appointment) or does the option of being able to see medical staff at [the SSP] during a drop-in seem more convenient for your lifestyle? Probe: Do you have a history of missing medical appointments? • When would be the best time of day for our team to engage individuals who are interested in LAI-PrEP? • Would you feel comfortable having it administered in our medical van, or would you want to come to the main site? Probe: How often have you been accompanied by SSP staff for medical appointments or visits to detox facilities or any type of care including shelter? • Is there anything else you would like to tell me that I did not ask you? • Do you have any last thoughts about the injectable PrEP and how it might work at IDEA?

**Table 2:** Qualitive interview questions with providers

Topics Addressed	Sample Question
Advantages of LAI-PrEP	• What are your current thoughts on LAI-PrEP? Probe: What are your thoughts on LAI-PrEP for PWID? • Would you feel comfortable referring PWID to the SSP for LAI-PrEP? Why or Why not?
Disadvantages of LAI-PrEP	• If someone stops or delays the injections, they will have to use alternative HIV prevention methods (condoms, oral PrEP, abstinence). • What are your thoughts on this? Probe: How often do you think you and/or your staff provide HIV prevention counseling to PWID?
Facilitators to LAI-PrEP	• At your organization, what are some of the things that make it easier for people who inject drugs (PWID) to get linked to care? Probe: Is this different for men and women? • At your organization, what things have you done/changes should be made to help PWID be able to obtain care? • What would be needed within your organization to help make this better regarding LAI-PrEP services?
Barriers to LAI-PrEP	• What are some of the ways that PWID are treated by others when they want to receive services? What suggestions do you have for making this better? Probe: Can you provide an example of mistreatment that you have witnessed? • At your organization, what are some of the *things that make it harder* for PWID to get linked to care? Probe: Is this different for men and women? • Do you have any concerns about a long-acting injectable PrEP, in general? • What are some of the barriers that PWID would have when they want to get LAI-PrEP treatment at a syringe service program? What suggestions do you have for overcoming these barriers?
Service Delivery Preferences	• What infrastructure would be needed at your organization to collaborate efficiently with the staff at a syringe services program? • Have you ever provided oral PrEP to PWID in the past? How were those experiences? Probe: What was most difficult about coordination of care? • What equipment, encryption, electronic health record and operating procedures would be necessary to ensure patient privacy and HIPAA compliance? • What is the legal framework, including liability, for proving remote care at a syringe services program? • What suggestions do you have for overcoming stigma for PWID within your organization?

**Table 3:** Client demographics (*n* = 25)

Demographics	*n* (%)
Age in years (median, range)	39 (43)
Sex assigned at birth	
Female	6 (24)
Male	19 (76)
Race	
Latino/a/x/e	8 (32)
Non-Latino White	11 (44)
Non-Latino Black	5 (20)
Indigenous American	1 (4)

**Table 4:** Provider demographics (*n* = 5)

Demographics	*n* (%)
Age in years (median, range)	35 (11)
Sex	
Female	3 (60)
Male	2 (40)
Race	
Latino/a/x/e	1 (20)
Non-Latino- White	2 (40)
Non-Latino - Black	1 (20)
Asian American	1 (20)
Duration of medical licensure in years (median, range)	4 (14)
Certifications	
Infectious Diseases	2 (40)
Internal Medicine	3 (60)
Family Medicine	1 (20)
Addiction Medicine	2 (40)
Critical Care Advanced Practice	1 (20)

### Data Analysis

The transcripts were analyzed using iterative thematic analysis with an a priori codebook drawn from the research questions and developed from the data using a general inductive approach. Client and provider transcripts were imported as separate projects in Dedoose (version 9.0.107, Sociocultural Research Consultants, Los Angeles, CA) to create respective codebooks and manage data methodically. To improve the quality of the research and ensure precise and comprehensive recording of findings, the consolidated criteria for reporting qualitative research (COREQ) checklist guided the reporting of study methods and results [25].

For the client data, three study team members reviewed separate transcripts and independently created an initial codebook. Next, the team met to integrate and refine the codebooks until consensus was reached. The codebook was further refined through the evolution of transcript analysis until saturation was achieved. For the provider data, three study team members analyzed the provider transcripts simultaneously and built on the client codebook to create a provider codebook agreed upon by all study members. Saturation was determined when no new codes emerged; this occurred with client data at transcript 17 of 25 and with provider data at transcript 3 of 5. Once codebooks stabilized, the remaining transcripts were coded collaboratively for completion. Any differences were negotiated to reach consensus on all transcripts (*n* = 30). All transcripts with inaudible or unintelligible language were audio-checked by study team members to ensure accuracy. Once all client and provider transcripts were coded, code application and co-occurrence were analyzed across projects. We synthesized findings from each group and holistically identified salient categories and themes.

## Results

### Acceptability

Overall, there was overwhelming consensus among both clients and providers regarding the acceptability of LAI-PrEP for PWID. Clients expressed enthusiasm for the possibility of LAI-PrEP implementation at the SSP, with statements such as “I think that’s incredible” (Client (C) 19), “this is something I would really, really appreciate” (C7), “I’m down for it 100%” (C18), “they should’ve had this a long time ago” (C20), and “I can’t really think of any negative things people would say about it” (C11). Providers were similarly supportive, as exemplified by the following: “In general, and for PWID, I think it’s a great development” (Provider (P) 3).

### Advantages

#### Challenges with Oral PrEP Adherence

All SSP clients expressed challenges taking oral PrEP due to factors related to their substance use and/or insecure living situations, which potentially could be mitigated with LAI-PrEP. They described difficulties storing the medication noting, *“It is a big problem. I have [to] leave my stuff somewhere every day – and*,* right now*,* I don’t have a purse…I have nowhere to carry it”* (C7). Another client expressed concerns regarding the effects of the Miami heat on PrEP medications, stating *“where I stay*,* I don’t have air conditioning*,* and you have to store it at a certain temperature”* (C21).

A particularly salient issue was the ubiquity of theft in the unhoused community: *“it’s just like a given when you’re out on the streets*,* people steal your stuff”* (C8). This is especially true of medications: *“They don’t care. It could be aspirin…if it’s a pill*,* they’re gonna think they can come up on it*,* they’re gonna steal your shit*,* man”* (C2). *“I’ve had my stuff stolen so many times”* (C11), lamented another, *“then [you] have to wait to see a doctor*,* and then*,* like*,* it might be on a weekend*,* so you didn’t even get your medicine”* (C3). Because of their substance use, many clients also described *“losin’ track of stuff”* (C11) and of time. One described *“pass[ing] out…if the fentanyl’s a little stronger than normal*,* or [you] do a little bit more*,* or different batch…next thing you know it’s like the middle of the night or the next morning”* (C8). *“The time and the day gets mixed up”* (C7), and *“a lotta us forget to do things*,* the simple things in life*,* like I said*,* eat*,* drink water*,* take medication”* (C20), making it difficult for PWID to adhere to a consistent medication schedule. One client stated: *“One thing about injecting drugs*,* shootin’ drugs*,* smokin’ it*,* sniffin’ it*,* one thing we do*,* and I’m speaking from experience. We forget to eat. We forget to drink. We forget to do a lotta things*,* hygiene. I can imagine forgetting to take a pill every day would probably be the last thing we’re probably thinkin’ about.*” (C20).

Consequently, many clients also described the mental load of *“keeping track”* (C11) of their medications and remembering to take oral PrEP every day, especially when compounded with the stresses of *“their chaotic lives”* (P4) and the effects of substance use. They referenced the hierarchy of needs, explaining that PWID are *“so focused on surviving and getting high”* (C19), and that *“the fear of getting sick is so great that it…surpasses everything*,* unfortunately”* (C7). When PWID have immediate concerns such as *“what am I gonna eat*,* where am I gonna sleep?”* (C21), they *“can’t really stop and think about*,* oh I gotta take this medicine”* (C21). One client noted, *“having that worry*,* burden*,* to take something every single day*,* know where it is and stuff like that*,* is a big deal”* (C7). Challenges of adherence to oral PrEP were also noted by all five providers. One stated simply, *“I have a hard time knowing if patients are taking it or not”* (P4). Another reflected, *“I think adherence is really poor*,* and I think people go on and off of PrEP all the time”* (P3). A third reiterated, *“getting [clients] here once a month to pick up their bottle of PrEP before they run out has been a big*,* big issue”* (P5).

#### Ability to Avoid Side Effects of Oral PrEP

A few clients reported that they experienced side effects from oral PrEP, which they thought might be reduced and/or avoided with LAI-PrEP. Nausea was the most reported symptom, and this was exacerbated by opioid withdrawal symptoms and inadequate nutrition, either due to *“not having food”* (C25) or having a diminished appetite due to the hot climate. As stated by one client, *“sometimes I literally dread taking that pill…if I already feel sick…there’s no way”* (C8).

#### Ease of Use of LAI-PrEP

In contrast to the challenges of oral PrEP, all the clients expressed excitement regarding the ease of use of LAI-PrEP, namely requiring only one injection every two months. They expressed enthusiasm regarding the convenience, specifically that it would *“cut down on things [they] have to keep track of”* (C5), and that they would *“not [have]…to keep up with the remembering to take it*,* the cost of the prescription*,* just the storing of it”* (C8). One client emphasized how meaningful this would be by stating, *“that’s fantastic. That would be wonderful. It would be a huge relief for many people”* (C7).

Importantly, when asked if they believed it would ease the burden of adhering to PrEP, one client responded, *“100%”* (C19). *“I’d have it in me*,* and I don’t have to worry”* (C23), said another. A third spoke candidly: *“I’m not gonna lie*,* I have missed maybe a day or two*,* or whatever. [But] doing that once every two months sounds so much better and easier. It sounds great”* (C18). This advantage was echoed by all five providers, especially given that *“we don’t have a lot of PrEP options [so] anything that’s a different modality of delivery is a beneficial option”* (P3). One reflected, *“I work with patients that will come in and tell you I ran out of my PrEP two weeks ago and I’d like to restart it*,* or I forgot to take it*,* or my medications have been stolen…I think anything that can be given long-term and doesn’t have to be something that requires the patient’s attention every day is of benefit in this patient population”* (P5).

Multiple providers described the increased confidence they would feel with regard to client adherence. As one provider described, *“You put it in. You know that they have it for a month. It’s…easier to ascertain from a physician’s perspective”* (P4). The same provider noted that this would also aid in their clinical decision-making, because then *“if the treatment doesn’t work*,* [we know] it’s not because of adherence issues”* (P4).

### Disadvantages

#### Side Effects of LAI-PrEP

The majority of clients (*n* = 21) noted that substantial side effects would dissuade them from taking LAI-PrEP. Participants who had taken oral PrEP in the past and experienced side effects (e.g., nausea, diarrhea) questioned whether they would also be present with LAI-PrEP. In particular, multiple clients worried that side effects would last for the full duration of the medication’s activity: *“If you do have side effects and it’s an injection that lasts two months…does that mean you’re gonna have it for two months?”* (C7). Another hypothesized,*“Obviously*,* if the medication lasts for two months*,* then the side effects are gonna be there for two months. That’s a long time to have to wait for the side effects to go away. Even if it’s something besides the nausea*,* like what if it’s headaches*,* or dizziness”* (C8). A third worried, *“If you have a bad physical reaction*,* then I say*,* guess what. It’s too late*,* It’s in your system. Now you gotta deal with it. It’s not like the medicine—you have a bad reaction and you might say*,* okay*,* I’m gonna chill out for a couple of days*,* see what happens. But PrEP—once you shoot it up*,* that’s it. If you start bouncing off the walls*,* guess what. You gotta bounce off the wall”* (C23).

This concern was well-recognized by providers, with one stating *“there’s always hesitancy about long-acting medications because once you give it you can’t take it back”* (P5). Because of its *“different form”*, clients also wondered if it might have *“different symptoms that may come with it”* (C18). Similarly, they reflected on previous negative side effects with other medications and wanted to avoid similar experiences in the future. Importantly, most of these clients were not aware of specific side effects or safety concerns associated with LAI-PrEP, but said they *“would wanna learn more”* (C11) before making a decision. However, one client concluded, *“if it’s been approved by someone*,* I’ll take it”* (C20).

#### Safety Concerns of LAI-PrEP

As with side effects, the majority of clients (n = 19) wanted to know if there were any safety concerns associated with LAI-PrEP. One asked about the possibility of infections at the injection site, stating *“I know when I shoot something into my arm*,* I usually end up with a spot from where*,* like*,* the dirt’s gotten in or whatever”* (C2). More commonly, clients wondered about potential interactions between LAI-PrEP and the substances they used: *“Now I’m shooting something that is foreign to what I’m usually doing. I don’t really know the reaction I’m gonna have since I’m doing other controlled substance”* (C23). “*We don’t know what chemicals don’t go together or shouldn’t go together … Everybody might have a different reaction. If I take it*,* it may not harm me at all*,* but someone else takin’ it*,* it may affect them based on their DNA. That’s what I feel”* (C20).

A few clients also cautioned that medical mistrust might pose a barrier to LAI-PrEP, explaining that some PWID may think *“maybe it’s something else that they’re injecting in you*,* maybe a virus or something…and be like*,* “No*,* I don’t want it”*(C3). One described not wanting to be *“on experiment mode…like a guinea pig”* (C23). Another worried about *“hear[ing] that the medication has done effect to others along the road that causes some kind of internal stimulation towards your liver or something like that”* (C9). Other than potential allergies, however, it is notable that no providers discussed significant safety concerns.

#### Fear of Injection

Paradoxically, the fact that LAI-PrEP is administered by injection was mentioned as a possible disadvantage by some clients. It was noted that people who don’t inject substances might be “*terrified of needles*” (C8), and even PWID may “*have issues with shots and needles and stuff*” (C5) and not “*want anything to do with anything to do with needles stickin’ in their arm or being tested anywhere*” (C20). One client asked about the pain of the injection, and another client reiterated “*scary*,* scary needles*” (C4). Healthcare providers corroborated this concern, stating “*even though we’re dealing with PWID*,* some of them are afraid of injections*” (P5). However, another provider said that while “*some people [who are afraid of injections] will not want it*,* if the people who do want it get it*,* that will be good*” (P4).

#### Risk of Viral Resistance

Finally, while all clients agreed that the ease of use of LAI-PrEP would facilitate greater adherence as compared to oral PrEP, most (*n* = 23) emphasized that barriers to adherence would still exist. Even clients who said they’d *“be pretty consistent”* acknowledged that this might mean *“from 0 to 100*,* like 90”* (C21). They cautioned that other things in their lives – *“waiting for a dealer*,*…[making] some money”* (C4), *“getting high”* (C23) – would still *“take precedence”* (C4). As one client noted *“you could be busy doing something*,* and*,* boom*,* you missed it”* (C23), and another said that while they might think *“I’ll go tomorrow*,* it’s one day*,* one day [can] turn into a week*,* then a week into a month”* (C8).

While one client expressed confidence that the two-month window of time in which they need to be given their injection is manageable, they were reassured that *“if I miss a couple of days of not getting it* (LAI PrEP injection)*…I’ll still be covered”* (C20). Others worried about the risk of resistance if they do not meet the window of time for injection: *“I think that could make it really dangerous for someone…If they miss a dose and then they contract [HIV]—in that missed time*,* the virus becomes resistant?”* (C21).

The implications of adherence challenges with LAI-PrEP for HIV resistance were discussed by all five providers. There was consensus that the *“downsides of it are a little bit unknown…at this point”* (P3). While they acknowledged that *“getting HIV while you’re on [either type of] PrEP can cause trouble*,* difficulty in diagnosis*,* and potential difficulty in treatment due to drug-resistance”* (P3), they questioned *“the theoretical versus the actual risk”* (P3), wondered with *“how many people has this been studied”* (P3), and hypothesized that LAI-PrEP is *“not necessarily associated with more HIV resistance”* (P4) than oral PrEP.

## Feasibility of LAI-PrEP Implementation

### Feasibility

Clients and providers shared statements that suggest implementation of LAI-PrEP for PWID would be feasible at an SSP, though there was consensus that the organization must overcome specific barriers and capitalize on facilitators to maximize client engagement and adherence.

### Barriers

#### Inconsistent Engagement with Clients and Gaps in Care

Providers recognized that inconsistent engagement with clients and gaps in care at the SSP would be barriers to client uptake of and adherence to LAI-PrEP. They cautioned that *“not every medication or service that [clients] need”* is offered at the SSP and that although they *“try very hard to provide telemedicine visits in as close to real-time … provider availability sometimes becomes an issue”* (P5). In addition, the fact that *“we’re not 24/7”* (P4) limits the SSP’s ability to meet clients’ needs after hours or on weekends. One client elaborated:

*“They’re open from this time to that time. They got a big*,* green gate out there. When that’s locked*,* you can’t get in”* (C20).

While the SSP strives to provide comprehensive care, these limitations suggest the need for complementary efforts to expand *“lower barrier entry for medical services outside of the needle exchange”* (P3), so that clients have access to care when it is not available at the SSP. Referring to the dichotomy of needs between male and female clients, one provider shared thoughts on IDEA’s ability to serve female clients, specifically: *“I feel like we don’t do a lot of women-specific health services…that’s a need that women have*,* especially contraception*,* and we’re not really meeting it. I think when people do become pregnant*,* there’s also a lot of gaps in care regarding access to physicians and services [for] HIV treatment and prevention and substance use disorder”* (P3).

#### Appointment Logistics and Costs

Some clients recalled incidents when appointment logistics interfered with their ability to receive healthcare services at the SSP. Speaking about the challenges that unhoused individuals face, one client shared that *“being homeless*,* it’s hard to keep an appointment”* (C17). One client described: *“Sometimes life just gets in the way; I miss the bus or the train isn’t coming on time. Sometimes it’s not even my fault. Then dealing with your family or friends or where you’re sleeping*,* that’s running you late”* (C3).

Others identified barriers such as scheduling a convenient appointment (C4), having access to transportation (C15 and C17), and experiencing long wait times once they arrived (C19) as factors that might prevent clients from making appointments and adhering to scheduled care. In addition, *“[clients] might not have a phone to call in and schedule an appointment”* (P1). It was also noted that clients’ “*transient*” (C13) or “*semi-nomadic lifestyle*” (C24) limits their ability to access the site. A provider expressed that *“getting them [to the SSP] once a month to pick up their bottle of PrEP before they run out has been a big*,* big issue”* (P5).

The cost of LAI-PrEP was mentioned as a barrier to implementation by the providers, with one stating *“we’ll have a 340B pharmacy associated with our SSP soon*,* but we are in a non-Medicaid expansion state so people don’t even have health insurance coverage”* (P2). Said another, *“I think the cost is going to be a major barrier”* (P2). A third provider mentioned expenses such as salaries for staff capable of performing blood draws and administering the injection, and *“having a refrigerator”* (P3). The same provider cautioned that, *“sometimes with these specialty medications*,* you have to call the mail-order specialty pharmacy and have it delivered for each dose*,* but that is definitely not going to be feasible. We would need to have the medications available [at the SSP] and figure out the billing some other way”* (P3).

#### Lack of Knowledge and Awareness Regarding LAI-PrEP

Nearly all clients (*n* = 21) were unaware of LAI-PrEP. As one client remarked, the interview was *“the first time I’ve ever heard of it”* (C7). Due to its recent approval and FDA approval, some of the providers at the SSP have *“never had direct experience”* (P1) administering the medication and are *“not [yet] familiar with the profile or the risks”* (P5). However, others felt comfortable and confident prescribing and administering it to their patients.

### Facilitators

#### Recognition of HIV Risk

Nearly all the clients (*n* = 23) recognized that behaviors associated with injection drug use increase their risk of acquiring and transmitting HIV. One provider remarked that *“[clients] are pretty savvy about how HIV is transmitted”* (P3) and *“recognize their risk”* (P1) due to injection drug use. This acknowledgement of their heightened HIV risk translated into support for PrEP in general as an HIV prevention method among clients, which could be extended to LAI-PrEP:

*“The PrEP is good because I’ve had a lot of run-ins. I’ve been stuck by needles and I’ve had encounters with people that do have HIV”* (C7).

“*I found out [about PrEP] through a friend a mine because she was getting it. I was like*,* ‘Cool. Let me do that too*,*’ because of my risk factors”* (C13).

*“I take [PrEP] for safety*,* you know*,* I might run into a needle*,* somebody could just be mean and want to stick me*,* or I [could] prick myself while putting my hand in the garbage”* (C14).

*“That person’s not taking their medication*,* we get stuck*,* or we have unprotected sex*,* or if [a condom] breaks ⁠—[PrEP] gives us the control to know that we’re gonna be safe”* (C7).

#### Characteristics of the SSP

All five providers identified the existence of the SSP in the community—as well as its culture of meeting PWID where they are and its goal of serving as a “one-stop-shop” to meet their various needs—as a facilitator for LAI-PrEP implementation. All five providers articulated that, by design, the SSP increases the accessibility of care for PWID in Miami. Notable facilitators cited by providers that could be leveraged to promote LAI-PrEP uptake and adherence include utilization of the SSP’s mobile unit, disease and overdose prevention resources, wrap-around mental and physical health services, the SSP’s destigmatizing environment, and its staffs’ commitment to clients’ well-being.

Clients emphasized that the one-stop-shop model of the SSP offers a *“safe haven”* (C7) for PWID in Miami, while also providing numerous services *“right in the community”* (C17). They considered it *“the mother of all needs”* (C4)–a convenient place where *“at the same time they come to exchange their needles … they can also [get medication]”* (C18). As another described, PWID are able to *“come to one place and get everything they need*,*”* (C4) without entering a formal healthcare setting.

Providers conveyed confidence that clients would consider LAI-PrEP if they recommended it, given their established foundation of trust and respect: *“They get it from a place of love where they are not going to be judged…They know that we’ve given our entire careers to them. They know that we care for them immensely and that we wouldn’t recommend anything that we wouldn’t recommend for a family member”* (P2).

Clients corroborated this and expressed gratitude for the dedication of the SSP’s staff and providers to their health and well-being. One client shared that it is *“a place to call home*” (C19). Another remarked, the *“staff is amazing here and*,* like I said*,* they have literally come to the street to deliver my medication to me”* (C7). A third recalled that an SSP staff member was the *“one face I remember in the hospital with my head injury and it’s nice because I no longer have family or anybody to visit me*,* so when I see you guys*,* it’s a friendly face”* (C7).

Finally, providers recognized that *“having a staff that is very culturally sensitive to this population is important”* (P3). The SSP staff and providers were described by clients as *“understanding”* (P3) and *“trusted providers”* (P1), and as people who *“won’t treat [PWID] with stigma”* (P3). This contrasts with providers in the formal healthcare system where there’s *“judgment*,*” “blame*,*”* and *“not a lot of compassion for people who are judged as putting themselves in this position”* (P4).

#### Incentives as a Tool for Engagement

One provider stated that compensation “*opens the door*” (P5) to increase uptake of HIV prevention interventions among this population. This belief was echoed by clients: *“Money is always a good incentive. It helps because in order from them to see they could benefit*,* they have to be lured in somehow*,* and I guess that’s the best way to do it”* (C21). Said a third, *“Compensation is always good encouragement*” (C11). Added a fourth, “*Paying them is gonna be ranking over everything*.” (C19). Over half of the clients reported that they would be willing to adhere to LAI-PrEP and stated that LAI-PrEP would be their preferred method of HIV prevention. However, they noted they would be more likely to adhere to care if they received monetary incentives.

### Recommendations for Implementation

Considering the barriers and facilitators to offering LAI-PrEP at the SSP described above, clients and providers proposed the following recommendations for implementation.

#### Increase Status-Neutral HIV Prevention Counseling

Clients emphasized a need to increase status-neutral HIV prevention counseling among PWID because *“the data about HIV in the area*,* it’s pretty scary looking”* (C2). Though clients reported *“learning more and more people have HIV in Miami”* (C24), they lamented that *“HIV isn’t high on everybody’s list”* (C4). However, from their perspective, increased status-neutral HIV prevention counseling at the SSP would help PWID understand “*that there’s a way to reduce the risk*” (C5) and *“would help all those who are on drugs because people who are not wanting to…catch HIV*,* they will spread the word around and share the knowledge”* (C9).

Providers described how their encounters with PWID could be adapted to include status-neutral HIV prevention counseling: *“If I’m seeing somebody for a wound infection*,* I’m not usually deliberately doing HIV counseling. Maybe that’s something that I should consider*,* just something to interject with every interaction”* (P5). They reflected that status-neutral HIV counseling could encompass a range of HIV prevention and harm reduction interventions, including “*safer-injection practices…using the same syringe only one time*,* not sharing syringes*,* not sharing other injecting equipment*,* using condoms when having sex*,* and using PrEP”* (P3).

There was consensus among both providers and clients that an important area of focus should be the risk of viral resistance if non-adherent to the LAI-PrEP dosing schedule. *“I just didn’t know HIV could become drug resistant at all”* (C11), clients responded when they learned about the risk of viral resistance. “*Really? Wow!*” (C14), said one; “*Oh*,* shit. Really?!*” (C19) reacted another. Providers emphasized the need to be *“completely upfront about the risks”* (P4) of viral resistance to help *“patients understand the importance of receiving the medication on a scheduled*,* regular basis”* (P5).

#### Increase Staff Coordination

The providers emphasized that administering LAI-PrEP within a certain “*window*” (P4) of time requires increased coordination *“compared to how we’re delivering regular PrEP which is maybe a little bit haphazard and scattered.”*(P3) While brainstorming possible ways to improve coordination, another provider suggested that they could “*call them—try and maybe have a separate list*,* like a panel of patients where I’m like*,* okay*,* these are the people that have started on LAI-PrEP. I need to make a note in my calendar to call them before the two months are up”* (P1).

The providers recommended that coordinating LAI-PrEP services for PWID would also require a *“mechanism for locating these patients every two months”* (P5); one suggestion provided was having *“the phlebotomist or the medical assistant go out on the van to give it to people when it’s due*” (P4). They also forecasted vertical job expansion: *“If we now start having a bunch of people that we need to find every two months*,* the workload increases. Like I said*,* I think the infrastructure is there”* (P5).

There was consensus that the SSP’s community engagement team, with their expertise in harmonizing the various services that PWID receive from the SSP, would be a main pillar of staff coordination efforts. A providers suggested using *“the medical records from the University to track when people are due*,* and the community engagement team saying*,* Hey*,* these are the people we’re looking out for this week”* (P4). Another recommended that “*with the help of [the community engagement team]*,* it’ll be easier to track them down. If a patient shows up earlier than their two months*,* I can assess them and remind them to show up at this time for their next injection”* (P1).

#### Improve Staff Capacity and Task Delegation

Team capacity planning emerged as an important area for modification among providers to optimize LAI-PrEP delivery to PWID at the SSP. These potential modifications included “*having someone with a medical-assistant level of training available”* (P3) who possesses *“the ability to do all the follow-up labs that are needed onsite*,* especially the HIV RNA”* (P3) and being able to troubleshoot scenarios, such as if *“someone’s a little bit outside of the window; should they get their next shot*,* or do something else? Some sort of on-call physician would probably have to be available to help make those decisions”* (P3). The emotional capacity of the staff was also considered: *“We need probably more staff so that people aren’t burnt out”* (P4).

Due to their multidisciplinary skill sets, staff efforts are currently distributed across various projects within the SSP. Thus, providers noted that *“we don’t always have an onsite provider”* [at the SSP] (P5) but “*try very hard to provide telemedicine visits in as close to real-time as we can”* (P5). With regard to LAI-PrEP, however, providers highlighted the importance of meeting people where they are and expressed willingness *“to do the injections…at the SSP physically more often”* (P1). However, they also suggested that some tasks could be delegated; for example, *“a nurse [could] administer it”* (P1). Likewise, telemedicine could be leveraged so *“the doctor follow-up doesn’t have to be on the same day or time as the injection”* (P3).

#### Optimize Appointment Availability & Logistics

Clients expressed a strong preference for drop-in appointments: *“A drop-in time would be more convenient”* (C5) said one; *“drop-ins are always a plus”* (C4) remarked another. However, they acknowledged that this might not be feasible, and they expressed a preference for appointments during the *“morning”* (C18) or *“mid-afternoon”* (C7). One client suggested that the SSP consider extending its hours of operation: *“The only barrier I know about this place here is the time (C20).* Another client indicated that staff members’ skill sets were more important than the location where services are provided: “*Honestly*,* it has nothing to do with where I feel more comfortable*,* it has to do with the person that’s administering [the injection]”* (C16).

Other suggestions to encourage PWID to attend their appointments included providing *“bus passes*,* train passes’’* (C24) and sending PWID who have phones “*reminders with text*,* or calls*,* or emails”* (C11). The most common recommendation among clients, though, was to leverage the SSP’s outreach capabilities through its mobile unit: *“If there was a van that came around*,* that would be awesome*,* that found me where I was sleeping”* (C3), and *“Well*,* if you guys could figure out a way to come to us as a mobile unit”* (C16). The possibility of leveraging the mobile unit for LAI-PrEP related services appeared to increase PWID confidence in their ability to adhere to the medication. Specifically, they expressed appreciation for *“being able to have that transportation available get to our medication no matter what”* (C7). Some clients already expected that the mobile unit would be utilized to increase coordination of care: *“Now you also got a van; you could go around”* (C23). Lastly, clients felt *“the medical van’s probably easiest”* (C8) when it came to locating PWID in need of medical care: *“Look*,* the van would be cool. If the van wants to come to me*,* that’d be great”* (C24).

## Discussion

The most salient and promising findings from this study were that all PWID interviewed – irrespective of age, gender, race, and ethnicity – were open to using LAI-PrEP and see the SSP as an ideal location for the delivery of this medication. It is important to note that the SSP from which the participants were recruited provides on-site access to oral PrEP, and all but one of the 25 clients interviewed had experience with oral PrEP (this individual reported taking PrEP during the pre-screening process, but later reported no previous experience with oral PrEP during the interview). This study adds an important perspective to the existing literature, as most PrEP-related studies conducted with PWID include individuals who lack previous experience with PrEP [13]. It is also noteworthy that most of the clients accessing oral PrEP at the SSP had never heard about the long-acting formulation prior to the interview. This finding highlights the importance of sharing up-to-date information about options for HIV prevention with PWID, especially as LAI-PrEP becomes more available and accessible.

Our study team believed that exploring the perspectives of clients already using oral PrEP regarding the acceptability of switching to LAI-PrEP might provide the insight needed to bolster the acceptability and feasibility of these services. Our iterative thematic analysis revealed that those who interacted with oral PrEP services at the SSP saw the implementation of LAI-PrEP as the logical next step for advancing HIV prevention care for the population. The importance of preventing HIV among PWID was a shared sentiment by all the providers and clients interviewed. Similar to other research studies on this topic [7, 8, 13, 26], interviewees recognized various structural challenges faced by PWID and elucidated upon how substance use disorders and homelessness serve as pervasive barriers to oral PrEP adherence. Clients shared a sense of hope that the injectable formulation of PrEP could help to overcome these challenges.

Regarding initiation of treatment, the results of this study suggest that syringe exchange is a crucial point of engagement when HIV prevention counseling can be administered by SSP staff. Those interested in LAI-PrEP could then be linked to a provider the same day. Following the initial administration of LAI-PrEP, the routine exchange of syringes facilitated by outreach workers and SSP staff could act as temporal engagement points where follow-up appointment reminders could be provided. Clients could also provide updates on their latest location and/or share a current working phone number. All participants reflected on how developing and applying sustainable protocols which ensure that PWID on LAI-PrEP maintain adequate therapeutic levels of cabotegravir would require synergy between clients, outreach workers, and providers.

The synergy involved in assisting PWID to obtain LAI-PrEP injections within a certain time frame after their initial dose (one month after the first injection, every two months thereafter) was addressed by participants, with a majority suggesting that SSP staff could facilitate meeting these intervals by increasing mobile operations and expanding hours of operation. Due to the LAI-PrEP guidelines recommending HIV RNA quantification every two months via blood draws and intramuscular injection of the medication [27], nursing or medical assistant staff would need to be embedded within these efforts. While these services are intensive, they could empower PWID, remove barriers to access, and increase the likelihood of LAI-PrEP adherence. Creating partnerships between traditional, standalone SSPs and community organizations such as Federally Qualified Health Centers, and leveraging the resources available to SSPs embedded within health departments could facilitate the SSP-based, low barrier medical services model suggested by the interviewees.

This study has a few limitations. All clients and providers were recruited from a single SSP embedded within an academic health center, so the opinions presented here may not all be generalizable to SSPs operating in different contexts. All clients interviewed were actively receiving services from the SSP, so social desirability bias may have affected client responses. PWID with HIV were not recruited, so the viewpoints of PWID who may have taken PrEP or may not have had the opportunity to take PrEP at some point prior to acquiring HIV are not represented. Finally, only clients who reported taking PrEP were included in this study, and this may have limited certain learnings that could have been derived from PrEP naïve individuals using the SSP’s services. Nonetheless, our research team used rigorous iterative thematic analysis to explore the views about the implementation of LAI-PrEP at an SSP with PWID and the providers who provide care for them, answering the call for more studies to evaluate the acceptability of LAI-PrEP among PWID and to explore implementation strategies for low barrier access to LAI-PrEP at SSPs [7, 10].

## Conclusion

Overall, this study adds to the growing evidence that provision of LAI-PrEP at SSPs is acceptable and feasible and holds promise in expanding access to and uptake of HIV prevention services among PWID, a critical goal in *Ending the HIV Epidemic*.

## Data Availability

Data and materials are available upon request to the corresponding author.
